# Genetic mechanisms of antimicrobial resistance identified in *Salmonella enterica, Escherichia coli*, and *Enteroccocus* spp. isolated from U.S. food animals

**DOI:** 10.3389/fmicb.2013.00135

**Published:** 2013-05-23

**Authors:** Jonathan G. Frye, Charlene R. Jackson

**Affiliations:** Bacterial Epidemiology and Antimicrobial Resistance Research Unit, Agricultural Research Service, U.S. Department of AgricultureAthens, GA, USA

**Keywords:** antimicrobial, resistant, multidrug-resistance, animals, foodborne, *Salmonella*, *E. coli*, *Enterococcus*

## Abstract

The prevalence of antimicrobial resistance (AR) in bacteria isolated from U.S. food animals has increased over the last several decades as have concerns of AR foodborne zoonotic human infections. Resistance mechanisms identified in U.S. animal isolates of *Salmonella enterica* included resistance to aminoglycosides (e.g., alleles of *aacC*, *aadA*, *aadB*, *ant*, *aphA*, and *StrAB*), β-lactams (e.g., *bla*_CMY−2, TEM−1, PSE−1_), chloramphenicol (e.g., *floR, cmlA, cat1, cat2*), folate pathway inhibitors (e.g., alleles of *sul* and *dfr*), and tetracycline [e.g., alleles of *tet*(A), (B), (C), (D), (G), and *tetR*]. In the U.S., multi-drug resistance (MDR) mechanisms in *Salmonella* animal isolates were associated with integrons, or mobile genetic elements (MGEs) such as IncA/C plasmids which can be transferred among bacteria. It is thought that AR *Salmonella* originates in food animals and is transmitted through food to humans. However, some AR *Salmonella* isolated from humans in the U.S. have different AR elements than those isolated from food animals, suggesting a different etiology for some AR human infections. The AR mechanisms identified in isolates from outside the U.S. are also predominantly different. For example the extended spectrum β-lactamases (ESBLs) are found in human and animal isolates globally; however, in the U.S., ESBLs thus far have only been found in human and not food animal isolates. Commensal bacteria in animals including *Escherichia coli* and *Enterococcus* spp. may be reservoirs for AR mechanisms. Many of the AR genes and MGEs found in *E. coli* isolated from U.S. animals are similar to those found in *Salmonella*. *Enterococcus* spp. isolated from animals frequently carry MGEs with AR genes, including resistances to aminoglycosides (e.g., alleles of *aac*, *ant*, and *aph*), macrolides [e.g., *erm(A), erm(B)*, and *msrC*], and tetracyclines [e.g., *tet*(K), (L), (M), (O), (S)]. Continuing investigations are required to help understand and mitigate the impact of AR bacteria on human and animal health.

## Introduction

### Antimicrobial resistance (AR) in bacteria

Antimicrobial compounds have been used to treat bacterial infections since the middle of the twentieth century. These compounds were highly successful in treating various diseases and were widely used in both human and veterinary medicine. However, resistance to these compounds was detected in target pathogens only a few years after initiation of therapeutic use in humans (Alanis, 2005). The selective pressure created by the use of antimicrobials was identified as a driving force behind the emergence of resistance which was genetically encoded, inherited by subsequent progeny of the resistant pathogens, and in some cases could be transferred horizontally even to distantly related bacteria [as reviewed by Linton (1977)]. Because of their efficacy in treating and preventing disease, antimicrobials were used widely in food animal husbandry, and were also found to promote the growth of some animals when fed to the animals at sub-therapeutic levels (Aarestrup and Wegener, 1999; Mathew et al., 2007). Over the past few decades, the number of human infections caused by antimicrobial resistant bacteria increased which can make treatment of these diseases more difficult [reviewed by Alanis (2005)]. Because of the genetic nature of resistance and the ability to select for resistant organisms through the use of antimicrobials, using these compounds in animals was considered a potential source of antimicrobial resistant bacteria which could be transmitted to humans (Aarestrup, 2005; Aarestrup et al., 2008; Shryock and Richwine, 2010).

### U.S. monitoring systems

Research into the impact of antimicrobial use in animals on human health has focused on determining the prevalence of resistance in bacteria isolated from animals and from human infections (Tollefson et al., 1998; Aarestrup, 1999, 2004; Bager et al., 1999a; McEwen and Fedorka-Cray, 2002). These studies were then followed by characterization of the mechanisms leading to resistance and determining if resistance found in human infections were likely to have first occurred in animals followed by transmission to humans. To achieve these goals, antimicrobial resistance (AR) monitoring programs have been initiated globally. In the U.S., the Centers for Disease Control and Prevention (CDC), the Food and Drug Administration (FDA), and the U.S. Department of Agriculture (USDA) established the National Antimicrobial Resistance Monitoring System (NARMS) to monitor changes in antimicrobial susceptibilities of zoonotic pathogens from human and animal clinical specimens, from healthy farm animals, and from raw product of food-producing animals at slaughter and processing (http://www.fda.gov/AnimalVeterinary/SafetyHealth/AntimicrobialResistance/NationalAntimicrobialResistanceMonitoringSystem/default.htm; Tollefson et al., 1998, 1999). Similar programs were developed in Canada, Mexico, the European Union, and in other locations (Martel et al., 2000; Barton et al., 2003; Aarestrup, 2004; Biedenbach et al., 2006; Hammerum et al., 2007). This information is shared globally to detect the emergence of resistance and the dissemination of antimicrobial resistant foodborne pathogens (http://www.who.int/foodborne_disease/resistance/agisar/en/index.html). Many of these programs also include epidemiological, microbiological, and molecular biology research projects to improve our understanding of the data collected.

### Background, definitions, and descriptions of basic mechanisms and genetics

In addition to determining the prevalence of resistant bacteria, research associated with the surveillance programs focuses on determining the mechanism leading to resistance. Typically, mechanisms of AR fall into three categories: (1) inactivation of the antimicrobial, (2) efflux or changes in permeability or transport of the antimicrobial, or (3) modification or replacement of the antimicrobial target (McDermott et al., 2003; Walsh, 2003; Boerlin and Reid-Smith, 2008; Foley and Lynne, 2008). Resistances are genetically encoded and can vary from mutations in endogenous genes, to horizontally acquired foreign resistance genes carried by mobile genetic elements (MGEs) like plasmids. Both point mutations and horizontally acquired genes can encode all three categories of resistance. Point mutations in a promoter or operator can result in the overexpression of endogenous genes such as an antimicrobial inactivation enzyme like the AmpC β-lactamase gene, or an efflux system like the *mar* locus (Van et al., 2000; Siu et al., 2003; Tracz et al., 2005). Point mutations in genes encoding antimicrobial targets can result in a resistant target, such as mutations to the gyrase gene leading to the expression of a fluoroquinolone-resistant gyrase enzyme (Eaves et al., 2004; Hopkins et al., 2005). Exogenous resistance genes encoded on plasmids, integrons, phage, and transposons can be horizontally transmitted by transformation, conjugation, or transduction and these foreign genes can encode all three mechanisms of resistance. This includes genes encoding enzymes that inactivate the antimicrobial, such as β-lactamases that cleave the four membered ring in β-lactams, genes which encode efflux systems like *tet*(A), genes encoding a modified version of the enzyme that is the target of the antimicrobial, such as *dfrA*, or genes encoding an enzyme that modifies the antimicrobial target like a ribosomal RNA methylase, such as *ermB* (Carattoli, 2001, 2009; Boerlin and Reid-Smith, 2008; Ajiboye et al., 2009). Analysis of these resistance mechanisms can then be used to determine the genetic relationship between resistances found in isolates from animals and humans. Because of the diversity of genetic elements that lead to AR, it may be possible to determine if resistances seen in bacterial isolates from human infections are closely related to those found in animal isolates, thus identifying animal sources of resistant bacteria in human infections that can be targeted in order to reduce human disease (Bager et al., 1999b; Aarestrup, 2000b; Boerlin, 2004).

### Impact on health and medicine in animals and humans

When first detected, AR in bacteria was relatively rare and new antimicrobial compounds were discovered or developed that were not susceptible to the resistance mechanisms that had arose (Alanis, 2005). However, resistance mechanisms to new antimicrobials can develop, or existing ones can emerge due to selective pressure from their use, leading to increasing resistance in human and animal isolates over time (Swaminathan et al., 2006; Frye and Fedorka-Cray, 2007; Gilbert et al., 2007; Frye et al., 2008, 2011). Increasing infections with antimicrobial resistant bacteria is also accompanied by a decrease in efficacy of treatment with these compounds (Alanis, 2005; Stoycheva and Murdjeva, 2006; Walsh and Fanning, 2008; Ajiboye et al., 2009). In addition, infections caused by antimicrobial resistant bacteria have also been shown to result in increased morbidity and mortality in humans and animals (Mundy et al., 2000; Alanis, 2005; Foley and Lynne, 2008; Ajiboye et al., 2009; Gebreyes et al., 2009; Huehn et al., 2010). The consequences of antimicrobial use are also caused by prophylactic treatment of animals to prevent infection or antimicrobials employed as growth promoters. Concerns about selective pressure caused by the utilization of sub-therapeutic growth promoting antimicrobials have led to precautionary restrictions and bans on these applications (DuPont and Steele, 1987; Aarestrup and Seyfarth, 2000; Aarestrup et al., 2001; Anthony et al., 2001; White et al., 2002, 2004; Stokes et al., 2008). In some cases these bans have appeared to result in increased animal illnesses and increased therapeutic use of some antimicrobials in animal husbandry, thus confounding the evaluation of these practices in preventing resistance and protecting human and animal health (DuPont and Steele, 1987; Aarestrup and Seyfarth, 2000; Aarestrup et al., 2001; Anthony et al., 2001; White et al., 2002, 2004; Stokes et al., 2008).

While the proportions of resistant bacteria have fluctuated from year to year, the percentage of antimicrobial resistant bacteria seems to be increasing as well as the fraction of bacteria that are multi-drug resistant (MDR; Devasia et al., 2005; Alcaine et al., 2007; Johnson et al., 2011a). MDR bacteria are troublesome because it is possible for a pathogen to be resistant to all of the antimicrobial compounds that are used to treat the infections it causes (Alanis, 2005). In addition, horizontally transferred mechanisms of resistance are often physically linked on genetic loci such as plasmids, integrons, and transposons (Poole and Crippen, 2009; Douard et al., 2010; Frye et al., 2011; Glenn et al., 2011, 2012; Lindsey et al., 2011a). These genetic elements often encode MDR and are capable of being transferred to sensitive bacteria, rendering a new bacterial host MDR through a single transfer event (Zhao et al., 2003; Poole and Crippen, 2009; Frye et al., 2011; Lindsey et al., 2011a).

## Salmonella

### *Salmonella* diseases, pathogenesis, and antimicrobial therapy

*Salmonella enterica* is a ubiquitous pathogen which can infect many host species and cause different diseases (D'Aoust, 1997; Lavigne and Blanc-Potard, 2008). Hosts can range from companion animals to food animals and humans, and disease symptoms can range from self-limiting gastroenteritis to invasive systemic infections with a high mortality rate (Lavigne and Blanc-Potard, 2008). Some serovars of *S. enterica* can survive in a wide variety of hosts and cause different diseases such as *Salmonella* Typhimurium, which causes no symptoms in adult poultry, can cause gastroenteritis in humans, or cause highly invasive systemic enteric fever in mice (Goldberg and Rubin, 1988; Chan et al., 2003; Lavigne and Blanc-Potard, 2008). Other serovars are host specific like *Salmonella* Typhi which is an obligate human pathogen that causes invasive Typhoid fever (Bhan et al., 2005), which is extremely rare in the U.S., but can be endemic in other parts of the world. Most other serovars of *Salmonella* usually cause only gastroenteritis in humans while some other serovars can potentially cause both gastroenteritis and invasive infections in humans. *Salmonella* is usually transmitted by contaminated food with contamination originating from animal products or feces, contaminated processing equipment, or contaminated food handlers (Goldberg and Rubin, 1988). Additionally, *Salmonella* is sometimes antimicrobial resistant which can result in difficulty treating infections (Fluit, 2005; Stoycheva and Murdjeva, 2006). Because of the impact on human health, zoonotic transmission, and ability to acquire AR, *Salmonella* has been chosen as the sentinel organism for foodborne disease and for AR monitoring (Tollefson et al., 1998, 1999; White et al., 2001, 2002).

In the U.S., *Salmonella* is estimated to cause over one million human infections each year (Scallan et al., 2011). Most of these infections result in gastroenteritis that resolves after a few days; however, some infections can be chronic or invasive, especially in the very young, the old, and groups of people with compromised immune systems (Alcaine et al., 2007). In these cases *Salmonella* infections may require antimicrobial treatment to prevent further morbidity or mortality (Alcaine et al., 2007). First line antibiotic treatment in the U.S. is typically a fluoroquinolone-like ciprofloxacin or a third generation cephalosporin β-lactam such as ceftriaxone, and folic acid pathway inhibitors are also available (Mandal, 1990; Guerrant et al., 2001; Hohmann, 2001; Habib, 2004; Parry and Threlfall, 2008). However, in children and pregnant women, treatment is usually limited to β-lactams due to fluoroquinolone's interference with cartilage formation; therefore resistance to β-lactams is a considerable concern in *Salmonella* (Parry and Threlfall, 2008). In cases where infection is caused by *Salmonella* resistant to first line treatments, alternative second line antimicrobials may be used, such as aminoglycosides, or folic acid pathway inhibitors like sulfisoxazole or sulfamethoxazole with or without trimethoprim (Guerrant et al., 2001). In MDR *Salmonella* infections, the last line treatments are usually the aminoglycoside, amikacin or the carbapenems, imipenem or meropenem which are administered intravenously. Due to observed increases in morbidity and mortality in antimicrobial resistant infections, it has been suggested that resistant *Salmonella* are more virulent than sensitive strains (Mundy et al., 2000; Alanis, 2005; Foley and Lynne, 2008; Ajiboye et al., 2009; Gebreyes et al., 2009; Huehn et al., 2010). However, research into this has been inconclusive, and some studies have demonstrated that resistance to some antimicrobials such as fluoroquinolones actually reduces virulence in *Salmonella* (O'Regan et al., 2010).

### Phenotypic antimicrobial resistance in *Salmonella* isolated in the U.S.

Antimicrobial resistant *Salmonella* have been isolated globally from human infections, clinically ill animals, healthy food animals, food animal products, and fresh produce. In the U.S., since 1997, NARMS has collected data on AR in *Salmonella* isolated from humans, and animals, including chickens, turkeys, cattle, swine, and their retail meat products. The percentage of resistant isolates from these sources has been presented in NARMS reports for each year from 1997 to 2009 (http://www.fda.gov/AnimalVeterinary/SafetyHealth/AntimicrobialResistance/NationalAntimicrobialResistanceMonitoringSystem/default.htm). Antimicrobial compounds used for susceptibility testing in *Salmonella* are listed in Table 1. Resistance to all of these antimicrobials was detected in isolates from humans during 1997–2009; however, only 26 human isolates were resistant to the first line treatment ciprofloxacin and only two human isolates were resistant to amikacin making it a possible treatment option (Table 1). However, substantial resistance to ceftriaxone, which is also often used as a first line treatment for salmonellosis, was detected in humans and increased from 1997 (0.5%) to 2003 (4.4%) (http://www.fda.gov/AnimalVeterinary/SafetyHealth/AntimicrobialResistance/NationalAntimicrobialResistanceMonitoringSystem/default.htm) and has remained fairly steady from 2004 to 2009 (average of 3.1%; Table 1) (Frye and Fedorka-Cray, 2007; Frye et al., 2008). A similar trend was observed for animal isolates, especially from cattle where resistance to ceftriaxone increased from 0% in 1997 to 21.6% in 2003 (Frye and Fedorka-Cray, 2007; Frye et al., 2008) and then leveled-off. Resistance to other antimicrobials varied between human and animal isolates, and especially by animal source of the isolates. In human isolates, resistance to tetracycline averaged 16.9%; in animal isolates resistance averaged 34.9% for all animals, and over 50% in turkey and swine isolates (Table 1). Sulfamethoxazole/sulfisoxazole resistance in human isolates was 15.3% and in animal isolates it was 19.9% for all sources. Resistance to streptomycin was also found in human and animal isolates at 14.4% in humans and 26.3% for all animals. Similarly, ampicillin resistance was 14.3% in human isolates and 16.0% in animal isolates. Resistance to chloramphenicol was detected in human (8.8%) and animal (7.3%) isolates; however, this drug is not usually used to treat human infections in the U.S. Levels of resistance to gentamicin was detected at 2.1% in isolates from humans and 6.5% from animals, and resistance to combined folate pathway inhibitors, trimethoprim-sulfamethoxazole, were 2.0% in human isolates and 1.4% in animal isolates, making it also suitable as a treatment in the U.S. (Guerrant et al., 2001).

**Table 1:** **Resistance to antimicrobials in *Salmonella enterica* isolated from human and animal sources by NARMS 1997–2009**.

**Number of isolates tested**	**Humans 23585**			**Chickens 14169**			**Turkeys 3941**			**Cattle 8284**			**Swine 4594**			**Animal total 30988**	
	**Resistant isolates**	**% Resistance**	**% Range**	**Resistant isolates**	**% Resistance**	**% Range**	**Resistant isolates**	**% Resistance**	**% Range**	**Resistant isolates**	**% Resistance**	**% Range**	**Resistant isolates**	**% Resistance**	**% Range**	**Resistant isolates**	**% Resistance**
**ANTIMICROBIAL (RESISTANCE BREAKPOINT)**																	
**Aminoglycosides**																	
Amikacin (MIC ≥ 64 μg/ml)	2	0.01	0.00–0.07	0	0.00	0.00–0.00	0	0.00	0.00–0.00	0	0.00	0.00–0.00	1	0.02	0.00–0.47	1	0.00
Gentamicin (MIC ≥ 16 μg/ml)	511	2.17	1.30–2.92	1046	7.38	4.27–17.80	732	18.57	12.92–25.40	174	2.10	0.00–3.86	56	1.22	0.00–2.70	2008	6.48
Kanamycin (MIC ≥ 64 μg/ml)	914	3.88	2.21–5.68	385	2.72	1.20–4.10	747	18.95	10.35–24.30	722	8.72	6.60–13.70	305	6.64	3.60–11.70	2159	6.97
Streptomycin (MIC ≥ 64 μg/ml)	3406	14.44	8.90–21.37	3342	23.59	19.32–30.50	1537	39.00	28.95–46.70	1759	21.23	12.50–28.70	1491	32.46	26.32–40.10	8129	26.23
**β-Lactams**																	
Amoxicillin-clavulanic acid (MIC ≥ 32/16 μg/ml)	829	3.51	1.00–5.28	1350	9.53	0.50–15.59	196	4.97	0.40–13.20	1027	12.40	2.505–21.00	96	2.09	0.00–4.50	2669	8.61
Ceftiofur (MIC ≥ 8 μg/ml)	738	3.13	0.46–4.50	1351	9.53	0.50–15.39	183	4.64	0.40–12.40	1021	12.32	0.00–21.58	93	2.02	0.00–4.50	2648	8.55
Ceftriaxone (MIC ≥ 4 μg/ml)	731	3.10	0.54–4.40	1336	9.43	0.50–15.60	176	4.47	0.04–12.40	1014	12.24	0.00–21.00	83	1.81	0.00–4.50	2609	8.42
Ampicillin (MIC ≥ 32 μg/ml)	3310	14.03	9.70–18.29	1905	13.44	9.40–17.00	824	20.91	10.40–38.80	1600	19.31	9.20–28.10	631	13.74	10.80–19.20	4960	16.01
**Folate pathway inhibitors**																	
Sulfamethoxazole/sulfisoxazole (MIC ≥ 512 μg/ml)	3616	15.33	9.90–27.70	1747	12.33	8.50–24.8	1254	31.82	24.30–38.00	1712	20.67	15.00–27.36	1459	31.76	25.10–37.00	6172	19.92
Trimethoprim-sulfamethoxazole (MIC ≥ 4/76 μg/ml)	467	1.98	1.39–2.33	62	0.44	0.00–1.20	90	2.28	0.80–4.20	226	2.73	1.50–4.86	57	1.24	0.00–2.70	435	1.40
**Chloramphenicol** (MIC ≥ 32 μg/ml)	2074	8.79	5.70–11.56	308	2.17	1.30–4.60	158	4.01	0.80–5.54	1359	16.41	4.20–25.10	450	9.80	7.70–15.17	2275	7.34
**Quinolones**																	
Ciprofloxacin (MIC ≥ 4 μg/ml)	26	0.11	0.00–0.36	1	0.01	0.00–0.01	0	0.00	0.00	0	0.00	0.00	0	0.00	0.00	1	0.00
Nalidixic Acid (MIC ≥ 32 μg/ml)	446	1.89	0.92–2.40	42	0.30	0.00–0.80	144	3.65	0.66–5.40	46	0.56	0.00–2.00	3	0.07	0.00–0.33	235	0.76
**Tetracycline** (MIC ≥ 16 μg/ml)	3984	16.89	11.60–21.68	3872	27.33	30.50–35.51	2221	56.36	45.80–73.80	2305	27.82	20.90–36.90	2409	52.44	43.10–62.83	10807	34.87

### Genetic mechanisms of antimicrobial resistance found in *Salmonella* U.S. animal isolates

#### Aminoglycosides

Aminoglycoside antimicrobials were first introduced into clinical application in the middle and last half of the twentieth century primarily to treat severe infections caused by Gram-negative bacteria in animals (Maurin and Raoult, 2001; Schwarz and Chaslus-Dancla, 2001; Schwarz et al., 2001). Their use in treatment of infections in food animals is limited due to both their toxic nature and the persistence of residual antimicrobial in the tissue of the animals. In swine, aminoglycosides including gentamicin, neomycin, or streptomycin have been used to treat intestinal diseases such as scours in weanling pigs and swine dysentery (Maurin and Raoult, 2001; Schwarz and Chaslus-Dancla, 2001). Gentamicin is also used to prevent or treat *Salmonella* or *E. coli* infections in poults. In some European countries, neomycin is used in combination with the macrolide antimicrobial, lincomycin, for treatment of mastitis in dairy cattle caused by *E. coli* and *Staphylococcus aureus* (De Oliveira et al., 2000). The synergistic effect of an aminoglycoside antimicrobial with an antimicrobial that targets the cell wall of enterococci such as a β-lactam like ampicillin or penicillin are also used in human medicine to treat enterococcal infections (Arias and Murray, 2012).

The aminoglycosides function by binding to the 30S ribosomal subunit inhibiting protein translation. *Salmonella* resistance to aminoglycosides is usually an enzymatic modification of the compound; however, in other bacteria, active efflux of the compound or enzymatic modification of the 16S rRNA subunit to prevent the aminoglycoside from binding to its ribosomal target can lead to resistance. Mechanisms of aminoglycoside resistances in U.S. *Salmonella* animal isolates are primarily due to acetyltransferases, phosphotransferases, and nucleotidyltransferases which modify and inactivate the aminoglycoside (Shaw et al., 1993; Ramirez and Tolmasky, 2010). There are two systems of nomenclature for these genes and some researchers have also used additional name modifications to indicate new alleles resulting in a complex set of names, many of which are synonymous. These have been expertly reviewed in several papers as well as their prevalence in *Salmonella* throughout the world (Ramirez and Tolmasky, 2010).

The aminoglycoside acetyltransferases are usually named *aac*, followed by a numeral in parentheses to designate the target of their enzymatic activity on the aminoglycoside molecule [e.g., *aacC(3′)*]. This can then be followed by a roman numeral to indicate the resistance phenotype, and then a letter to indicate the allele or variant of the gene. The *aac* genes found in U.S. *Salmonella* isolates can confer resistance to gentamicin, tobramycin, and kanamycin. Aminoglycoside phosphotransferases confer resistance to kanamycin and neomycin, and are usually named *aph*. These genes also have a designation of the location they modify on the antibiotic [e.g., *aph(3′)*], and some *aph* genes also have other names, such as *strA and strB* which encode resistance to streptomycin. Aminoglycoside nucleotidyltransferases can confer resistance to gentamicin, tobramycin, or streptomycin and include *aad* and *ant* groups of genes that can also have extensions to indicate the target of the enzyme. The most common genes reported are listed in Table 2, and include variants of *aac*, *aad*, *aph*, and *str* genes. Specific alleles of aminoglycoside resistance genes detected in several studies of NARMS U.S. food animal isolates have included *aac(3′), aac(6′), aadA, aadA1, aadA2, aadA12, aphAI, aph(3′)-Ii-iv, strA*, and *strB* (Frye and Fedorka-Cray, 2007; Frye et al., 2008, 2011; Glenn et al., 2011; Lindsey et al., 2011a).

**Table 2:** **Antimicrobial resistance genes found in *Salmonella enterica* isolated from U.S. food animals**.

**Antimicrobial class**	**Genes**	**References**
Aminoglycosides	*aacC(3′), aacC(3′)-IIa, aacC(6′), aacC2, aadA, aadA1, aadA2, aadA12, aadB, ant(3″)-Ia, aphAI, aphAI-IAB, aph(3′)-Ii-iv, aph(3′)-IIa, strA, strB*	Foley and Lynne, 2008; Ramirez and Tolmasky, 2010; Chen et al., 2011; Frye et al., 2011; Glenn et al., 2011; Folster et al., 2012
β-lactams	*bla*_CMY−2_*, bla*_PSE−1_*, bla*_TEM−1_	Li et al., 2007; Foley and Lynne, 2008; Frye et al., 2011; Glenn et al., 2011;
Chloramphenicol	*floR, cmlA, cat1, cat2*	Foley and Lynne, 2008; Frye et al., 2011; Glenn et al., 2011
Fluoroquinolones	Mutations in Quinolone Resistance Determining Regions (QRDR) of *gyrA, gyrB, parC, parE*	Hopkins et al., 2005
Folate pathway inhibitors	*sul1, sul2, sul3, dfr1, dfrA10, dhfrI, dhfrXII*	Zou et al., 2009; Frye et al., 2011; Glenn et al., 2011
Tetracyclines	*tet*(A), *tet*(B), *tet*(C), *tet*(D), *tet*(G), and regulator *tet*R	Roberts, 2005; Foley and Lynne, 2008; Zou et al., 2009; Frye et al., 2011; Glenn et al., 2011

#### β-lactams

Penicillin was one of the first β-lactams developed for clinical use in humans, and was also one of the first antibiotics to which bacteria became resistant. The β-lactams prevent synthesis and maintenance of the peptidoglycan component of the bacterial cell wall by mimicking one of the building blocks used by enzymes to construct peptidoglycan (Prescott, 2000c,d,g). The β-lactams have a unique four membered “β-lactam” ring that when acted upon by enzymes that build the cell wall, forms an irreversible bond to the enzyme, inactivating it and preventing the enzyme from completing cell wall synthesis. These enzymes are also known as penicillin-binding proteins (PBP). Most resistance to β-lactams is conferred by β-lactamases that enzymatically cleave the β-lactam ring and prevent it from bonding to and inactivating cell wall enzymes (Prescott, 2000c,d,g). Because of this, new β-lactams were synthesized through modification of the chemical groups around the β-lactam ring to produce β-lactams that are resistant to the β-lactamases; other modifications also improved their activity on specific bacteria or accessibility to certain infection sites. These include modified penicillins such as methicillin and oxacillin; the cephalosporins like cephalothin, cefoxitin, ceftriaxone, and cefipime, which are 1st through 4th generation cephalosporins, respectively; and the carbapenems such as imipenem and meropenem (Prescott, 2000c,d,g). In response to the selective pressure created by these new antibiotics, mutations in β-lactamase genes have also created enzymes that can digest these later generation β-lactams. Some important groups of these are the extended spectrum β-lactamases (ESBLs) (Bush, 2008), cephalosporinases (Arlet et al., 2006), and carbapenemases (Miriagou et al., 2010). However, some β-lactamases can also be inactivated by β-lactamase inhibitors, like clavulanic acid, which bind irreversibly to particular β-lactamases, thus allowing the β-lactam to work when the β-lactam and β-lactamase inhibitor are used as a combined treatment, such as Augmentin (ampicillin/clavulanic acid; Prescott, 2000g). Other resistance mechanisms include genes that encode modified PBPs that have a low affinity for β-lactams and are not inactivated by them or that use different building blocks to construct the cell wall. Efflux of the β-lactam or modification of porins (e.g., *ompF* and *ompC*) is also a resistance mechanism to β-lactams. Often these different mechanisms are found in the same bacterium, resulting in high level β-lactam resistance (Batchelor et al., 2005).

Most of the β-lactam resistance in *Salmonella* is encoded by horizontally acquired β-lactamases (Table 2); however, many other bacteria have an intrinsic β-lactamase, such as *ampC* found in *E. coli* (Table 3) (Siu et al., 2003). In *Salmonella* isolated from U.S. animals, the most prevalent β-lactamase genes are *bla*_TEM−1_ and *bla*_PSE−1_ (a.k.a. *bla*_CARB2_) encoding resistance to ampicillin, and *bla*_CMY−2_ that encodes resistance to ampicillin, 1st, 2nd, and 3rd generation cephalosporins and is also resistant to β-lactamase inhibitors such as those found in Augmentin (Table 2) (Frye and Fedorka-Cray, 2007; Frye et al., 2008, 2011; Glenn et al., 2011; Lindsey et al., 2011a). In contrast, other β-lactamases have been detected globally including *bla*_TEM_, *bla*_CTX−M_, *bla*_IMP_, *bla*_VIM_, *bla*_KPC_, *bla*_SHV_, and *bla*_OXA_ and variants of these genes may encode ESBL or carbapenemase activity (Falagas and Karageorgopoulos, 2009). Of particular concern may be the global emergence of the *bla*_NDM−1_ metallo-β-lactamase that confers resistance to carbapenems which are often the last line of defense in Gram negative infections (Walsh, 2010). Recently this gene was found in *Salmonella* isolated from a human in the U.S. after travel to India (Walsh, 2010; Savard et al., 2011). NARMS U.S. food animal isolates of *Salmonella* collected from 1999 to 2011 have been screened by both phenotypic and genotypic assays for ESBLs and none have been detected in isolates from U.S. animals by these or any other published studies [(Frye and Fedorka-Cray, 2007; Frye et al., 2008) and Fedorka-Cray, pers. communication]. The β-lactam resistance genes identified in NARMS animal isolates have been limited to mostly *bla*_TEM−1_, *bla*_PSE−1_, and *bla*_CMY−2_ (Table 2) (Frye and Fedorka-Cray, 2007; Frye et al., 2008, 2011; Glenn et al., 2011; Lindsey et al., 2011a).

**Table 3:** **Antimicrobial resistance genes found in *Escherichia coli* isolated from U.S. food animals**.

**Antimicrobial class**	**Genes**	**References**
Aminoglycosides	*aac(3′), aac(6′), aadA, aadE, strA/B, aph*	Ramirez and Tolmasky, 2010; Frye et al., 2011; Glenn et al., 2012
β-lactams	*ampC, ampR, bla*_*CMY*−2_, *bla*_PSE−1_, *bla*_TEM−1_	Li et al., 2007; Frye et al., 2011; Glenn et al., 2012
Chloramphenicol	*cat1, cmlA, floR*	Keyes et al., 2000; White et al., 2000; Bischoff et al., 2002; Frye et al., 2011; Glenn et al., 2012
Fluoroquinolones	Mutations in Quinolone Resistance Determining Regions (QRDR) of *gyrA, gyrB, parC, parE*	Hopkins et al., 2005
Folate pathway inhibitors	*sul1, sul2, sul3, dfr*	Graves et al., 2002; Ajiboye et al., 2009; Frye et al., 2011; Glenn et al., 2012
Tetracyclines	*tet*(A), *tet*(B), *tet*(C), *tet*(G), *tet*(M), and regulator *tet*R	Bryan et al., 2004; Frye et al., 2011; Glenn et al., 2012

#### Phenicols

Chloramphenicol and related compounds such as florphenicol inhibit protein synthesis by binding to the 50S ribosomal subunit. In the developed world, chloramphenicol is virtually irrelevant clinically and has been banned in the U.S. and other countries for use in humans or food animals due to its potential toxic effects on humans. In most of the developing world, its use is also limited by high levels of resistance likely due to the low-cost of the antimicrobial and unregulated, widespread over use. It is primarily used for treatment of systemic salmonellosis caused by bacteria that are resistant to other drugs of choice (Prescott, 2000e). Chloramphenicol has also been used for eye infections and sparingly for treatment of infections caused by bacterial anaerobes. Most resistance mechanisms are efflux pumps such as *floR* and *cmlA*, as well as inactivating enzymes such as chloramphenicol acetyltransferase, *cat1* (Prescott, 2000b,e). Resistance in *Salmonella* and other bacteria isolated from animals is often seen by these mechanisms. Mechanisms of phenicol resistance found in U.S. NARMS animal isolates have been *floR*, *cmlA*, and *cat1* (Table 2). In addition, the chloramphenicol resistance gene *floR* is often found in the class I integron located in Salmonella Genomic Island 1(SGI-1) (Frye et al., 2011; Glenn et al., 2011, 2012).

#### Quinolones

Quinolones and fluoroquinolones are a synthetic group of antimicrobials used in food animals to combat various infectious agents (Hopkins et al., 2005). Introduced into use over two decades ago, they have broad-spectrum activity coupled with low toxicity and other pharmacokinetic characteristics which make them attractive antimicrobials for use in food animals. A number of fluoroquinolones have been used in food animals including enrofloxacin, difloxacin, marbofloxacin, orbifloxacin, and sarafloxacin (Hopkins et al., 2005). Enrofloxacin and danofloxacin are both useful for treatment of respiratory track disease in cattle; enrofloxacin and sarafloxacin were approved in the mid-1990's for treatment of chickens and turkeys with *E. coli* infections (Endtz et al., 1991; Nelson et al., 2007). Although valuable for treatment of disease in food animals, fluoroquinolones are also used in human medicine for the treatment of *Salmonella, E. coli*, and other bacterial infections. Bacteria resistant to fluoroquinolones used in animals could also be resistant to fluoroquinolones used in human medicine due to the shared mechanism of action of these drugs (Nelson et al., 2007). In Europe, after the introduction of enrofloxacin in food animals, ciprofloxacin-resistant *Campylobacter jejuni* were detected in food animals and humans and were suspected to have been transmitted through food; this led to EU withdrawal of use of these compounds (Endtz et al., 1991; Nelson et al., 2007). In the U.S., fluoroquinolone use in poultry is banned and use in cattle is limited to non-lactating heifers under 20 months of age for treating bovine respiratory disease in cattle.

Quinolones and fluoroquinolones bind to and prevent DNA processing enzymes such as topoisomerases from assisting in DNA replication and maintenance. Mechanisms found in *Salmonella* and *E. coli* have been recently reviewed (Hopkins et al., 2005). Most resistances to these compounds are due to mutations within the genes that encode the enzymes such as *gyrA*, *gyrB*, *parC*, and *parE*. Most of these mutations occur in the quinolone resistance determining region (QRDR) which is a conserved site in these enzymes targeted by these antimicrobials. Resistance to nalidixic acid and then to fluoroquinolones builds in a stepwise process of mutations in the QRDR region producing an enzyme with a target region that quinolones cannot bind to (Chen et al., 2007). Resistance to nalidixic acid and fluoroquinolones like ciprofloxacin has been found in human isolates of bacteria globally. However, animal isolates of *Salmonella* in the U.S. have very low levels of resistance while the close relative *E. coli* has higher levels of resistance; for example, only a handful of *Salmonella* isolated from animals by NARMS were resistant to ciprofloxacin (Tankson et al., 2006). Studies have shown that *Salmonella* resistant to ciprofloxacin also had a growth defect *in vitro* and *in vivo*, while *E. coli* does not (O'Regan et al., 2010). This may be responsible for the low levels of ciprofloxacin resistance seen in *Salmonella*. Other resistance mechanisms have also been identified including the *qnr* efflux system, and an aminoglycoside acetyltransferase, *aac(6′)-Ib*, which can modify and inactivate ciprofloxacin (Cavaco et al., 2007a,b; Cavaco and Aarestrup, 2009). While these mechanisms have been found in *E. coli*, they are rare or undetected in U.S. *Salmonella* isolates (Tables 2, 3) (Frye and Fedorka-Cray, 2007; Frye et al., 2008, 2011; Glenn et al., 2011, 2012; Lindsey et al., 2011a).

#### Folate pathway inhibitors

These are compounds that compete for substrates of the essential folic acid pathway in bacteria at two different steps, the sulfonamides, which inhibit DHPS (dihydropteroate synthase) and trimethoprim, which inhibit DHFR (dihydrofolate reductase). Sulfonamides are one of the oldest classes of antimicrobials introduced into use more than 80 years ago (Skold, 2001). Both sulfonamides and trimethoprim act on the folic acid pathway in bacteria by interfering with the production of dihydrofolic acid. They have been used extensively in food animals as growth promoters in swine and for treatment of diseases such as colibacillosis in swine and coccidiosis in poultry (Prescott, 2000b,h). Sulfonamides are bacteriostatic when used alone or bacteriocidal when used in combination (trimethoprim-sulfamethoxazole; Walsh, 2003).

Resistance to both of these antimicrobials occurs by acquisition of genes encoding enzymes that do not bind these compounds. These include the *sul* genes, *sul1*, *sul2*, and *sul3*, which encode an insensitive DHPS enzyme and are found in *Salmonella* globally; however, in the U.S. most of the resistance is due to either *sul1* or *sul2* (note that sometimes roman numerals are used to designate these genes; Prescott, 2000h). Resistance to trimethoprim is by DHFR encoding genes, either *dhfr* or *dfr*, both of which have been found in *Salmonella* animal isolates in the U.S. (Prescott, 2000h; Frye et al., 2011; Glenn et al., 2011). More than 30 *dfr* genes have been identified and are presently divided into two families (White and Rawlinson, 2001). Type A dihydrofolate reductase genes encode proteins between 157 and 187 amino acid residues, while type B dihydrofolate reductase genes encode proteins of ~78 residues (White and Rawlinson, 2001). Sulfonamides and trimethoprim are often used in combination to treat *Salmonella* infections that are resistant to other antimicrobials (Hohmann, 2001). Currently, resistance to these combined compounds is relatively rare in the U.S. as reported by NARMS and is a good second line treatment for salmonellosis (Table 1). Genes that have been identified in studies of NARMS *Salmonella* animal isolates include: *sul1* (a.k.a. *sulI*), *sul2* (a.k.a. *sulII*), *dfr1, dfrA, dfrAI, dhf*, and *dhfrI* (Table 2) (Zou et al., 2009; Frye et al., 2011; Glenn et al., 2011).

#### Tetracyclines

Tetracycline has been used in food animals to combat vector-borne infections such as borreliosis, erlichiosis, rickettsiosis, and tularemia as well as other infections including pneumonia, brucellosis, and listeriosis (Roberts, 1996, 2002, 2005). In food animals, tetracycline or doxycycline are used mainly in treatment of respiratory infections (Mathers et al., 2011). Tetracyclines (chlortetracycline or oxytetracycline) have also been used for growth promotion and to promote feed efficiency in cattle, swine, and poultry. Tetracycline targets the 30S subunit of the bacterial ribosome binding to the ribosome and inhibiting protein synthesis. Resistance mechanisms include efflux, modification of the rRNA target, and inactivation of the compound. However, in *Salmonella*, active efflux systems are most commonly observed and include *tet*(A), *tet*(B), *tet*(C), *tet*(D), *tet*(G), and *tet*(H). In U.S. *Salmonella* animal isolates, *tet*(A), *tet*(B), *tet*(C), *tet*(D), and *tet*(G) are most often detected and are usually accompanied by the *tetR* regulator (Frye and Fedorka-Cray, 2007; Frye et al., 2008, 2011; Zou et al., 2009; Glenn et al., 2011; Lindsey et al., 2011a). As reported by NARMS, tetracycline resistance is 16.9% in human isolates and 34.9% in animal isolates of *Salmonella* in the U.S. (Table 1). Tetracycline resistance has been linked to use over a long time in humans, in animals, and as growth promoters in animals (Witte, 2000; Chopra and Roberts, 2001; Jones and Ricke, 2003). The tetracycline resistance genes found most often in studies of NARMS *Salmonella* animal isolates are *tet*(A), *tet*(B), *tet*(G), and *tetR* (Table 2) (Frye and Fedorka-Cray, 2007; Frye et al., 2008, 2011; Glenn et al., 2011; Lindsey et al., 2011a).

### Genetic elements associated with antimicrobial resistance and multi-drug resistance in U.S. *Salmonella* food animal isolates

AR genes are often arranged in cassette-like genetic elements, which may include multiple resistances and be associated with integrons or MGEs that can facilitate the expression of these genes as well as their movement both within a bacterium's genome and horizontally between bacteria (Carattoli, 2001, 2003, 2008, 2009; Blake et al., 2003; White et al., 2003; Lindsey et al., 2011a; Douard et al., 2010; Folster et al., 2011; Frye et al., 2011; Glenn et al., 2011, 2012; Johnson et al., 2011b; Lindsey et al., 2009). These cassette-like elements may be associated with insertion sequences (IS elements), integrons, and MGEs like transposons, bacteriophage, and others. These cassettes may also be located on plasmids, which are self-replicating, extra chromosomal DNA elements that can also encode machinery for DNA transfer between cells such as conjugation systems (Carattoli, 2003, 2009). On a global level, *Salmonella* harbor a wide variety of these elements (Carattoli, 2003, 2009). These vary greatly with the location or host source of the isolates and often reflect the environment to which they are exposed (Carattoli, 2008, 2009). In *Salmonella* isolated from U.S. farm animals, the most prevalent genetic elements identified to date are plasmids and integrons (White et al., 2003; Lindsey et al., 2009, 2011a; Folster et al., 2011; Frye et al., 2011; Glenn et al., 2011, 2012; Johnson et al., 2011b).

Plasmids are categorized by incompatibility groups, or Inc types, as only one plasmid of the same type can be stably maintained during cell division; thus two plasmids of the same Inc type are “incompatible.” Recently replicon typing has been accepted as an improved plasmid typing method using specific PCR assays (Carattoli et al., 2005, 2006). Replicon typing further divides Inc groups into sub-groups of replicons based on their specific DNA sequence. Therefore, replicon type is not necessarily interchangeable with Inc type (Carattoli et al., 2005, 2006). Studies of antimicrobial resistant, including MDR, *Salmonella* have determined that specific plasmid replicon types are associated with resistance, source host, and geographic origin (Osborn and Boltner, 2002; Su et al., 2004; Carattoli, 2008, 2009; Lindsey et al., 2009, 2011a,b). In U.S. food animals, isolates harbor multiple replicon types, including IncFIB, IncFIIA, IncHI1, IncHI2, IncI1, IncA/C, and IncP (Lindsey et al., 2009, 2011a; Glenn et al., 2011). Because IncA/C plasmids are wide spread in U.S. isolates and often carry MDR, they have been well-studied (Welch et al., 2007, 2009; Fricke et al., 2009; Lindsey et al., 2009, 2011a; Glenn et al., 2011).

Integrons are also associated with AR. These are inserted into the host bacterium's genome or plasmids, have an integrase gene, have cassettes and promoters bounded by IS elements, can mobilize their cassettes, and may have a variable number of genes inserted into the cassettes, including AR genes. While there are also several kinds of integrons detected in *Salmonella* isolated from animals in the U.S., the most prevalent is IntI1 and its variants, which often encode several AR genes and consequently may confer MDR phenotypes (White et al., 2003; Ebner et al., 2004; Gebreyes et al., 2004; Evershed et al., 2009; Krauland et al., 2009; Frye et al., 2011; Glenn et al., 2011; Lindsey et al., 2011a).

The development and dissemination of MDR in *Salmonella* is a major concern and the IntI1 integrons and the IncA/C plasmids play a major role in MDR found in isolates from U.S. food animals (Carattoli, 2001; Carattoli et al., 2002a,b; Welch et al., 2007, 2009; Carattoli, 2008, 2009; Fricke et al., 2009; Douard et al., 2010; Mataseje et al., 2010; Frye et al., 2011; Glenn et al., 2011; Lindsey et al., 2011a). *Salmonella enterica* serovar Typhimurium Definitive Phage Type 104 (DT104) was a MDR *Salmonella* that affected humans and animals worldwide in the mid 1990's (Wall et al., 1994; Akkina et al., 1999; Bolton et al., 1999; Briggs and Fratamico, 1999; Baggesen et al., 2000; Allen et al., 2001). It was first detected in Europe in the 1990's and was eventually found in U.S. swine and humans (Wall et al., 1994; Akkina et al., 1999; Bolton et al., 1999; Briggs and Fratamico, 1999; Baggesen et al., 2000; Allen et al., 2001). DT104 was reported as penta-resistant to ampicillin, chloramphenicol, streptomycin, sulfamethoxazole, and tetracycline (ACSSuT); however, isolates were subsequently detected with different phenotypes. The resistance genes usually associated with DT104 are *bla*_PSE−1_, *floR*, *aadA2*, *sulI*, *dfrA10*, *tet*(G), and *tetR* located on an IntI1integron (Boyd et al., 2001; Ebner et al., 2004; Mulvey et al., 2006). IntI1 is site specific and usually integrates into the genome resulting in *Salmonella* Genomic Island 1 (SGI1) a wide spread MDR carrying MGE (Ebner et al., 2004; Mulvey et al., 2006). In the U.S., penta-resistant *Salmonella* Typhimurium DT104 is found primarily in swine but has also been isolated from other animal sources including chickens, turkeys, and cattle. These integrons can also be found in other serovars of *Salmonella* and other species of bacteria, and can encode different resistance genes for multiple antimicrobials, resulting in many variants of IntI1 and SGI1 (Mulvey et al., 2006).

Recently, MDR *Salmonella enterica* serovar Newport was isolated from animals and humans with a MDR-AmpC phenotype which is similar to the ACSSuT DT104 phenotype, but also includes resistance to 3rd generation cephalosporins and sometimes additional antimicrobials (Gupta et al., 2003; Varma et al., 2006; Lindsey et al., 2009, 2011a; Glenn et al., 2011). As described previously, the β-lactam often used to treat human *Salmonella* infections is the 3rd generation cephalosporin, ceftriaxone, and resistance to ceftriaxone increased from 1998 to 2003 in humans and animals (Table 1). Several studies have suggested that this increase was associated with the use of ceftiofur, which is similar to ceftriaxone, in dairy cattle to treat mastitis and diarrhea (Clegg et al., 1983; Zhao et al., 2003; Cobbold et al., 2006; Lindsey et al., 2009; Glenn et al., 2011). This use may have exerted selective pressure causing MDR *S. enterica* serovar Newport to increase in prevalence. However, direct evidence for increased prevalence has not been reported (Cobbold et al., 2006; Call et al., 2008; Glenn et al., 2011). Ceftriaxone and ceftiofur resistance in MDR-AmpC isolates was determined to be encoded by the *bla*_CMY−2_ β-lactamase gene and was located on an IncA/C plasmid found in *S.* Newport isolated from U.S. humans and cattle (Gupta et al., 2003; Varma et al., 2006). These IncA/C plasmids were demonstrated to often be mobile, found in many serovars other than *S*. Newport, and also encoded resistance to multiple antimicrobials including other β-lactams, aminoglycosides, chloramphenicol, folic acid pathway inhibitors, and tetracycline (Fricke et al., 2009; Lindsey et al., 2009; Welch et al., 2009; Douard et al., 2010; Folster et al., 2011; Glenn et al., 2011; Lindsey et al., 2011a). Genes encoding the resistance mechanisms found in U.S. animal isolates often include *bla*_CMY−2_, *aacC*, *aadA*, *aphA1*, *strB/A*, *floR*, *cat*, *sulI*, *dhfr1*, *tet*(A), and *tetR* (Table 2) (Glenn et al., 2011). Several studies have shown that the resistance cassettes found in the IncA/C plasmids are similar to those in SGI1, and that these genes were likely previously transferred to the SGI1integron from an IncA/C plasmid in the distant past, prior to human influences (Fricke et al., 2009; Lindsey et al., 2009; Welch et al., 2009; Douard et al., 2010; Folster et al., 2011; Glenn et al., 2011; Lindsey et al., 2011a). It has also been demonstrated that IncA/C MDR plasmids can mobilize *SGI1 in trans* (Douard et al., 2010).

Interestingly, throughout most of the rest of the world, resistance to 3rd generation cephalosporins in *Salmonella* is often encoded by ESBLs, which are mutations of several different lineages of β-lactamases (Bradford, 2001; Paterson, 2006). The ESBLs can be found in *Salmonella* isolated from both animals and humans in Europe, Asia, and South America (Bradford, 2001; Nijssen et al., 2004; Hasman et al., 2005; Yates and Amyes, 2005; Biedenbach et al., 2006; Carattoli, 2008). *Salmonella* with ESBLs are infrequently isolated from humans in the U.S.; however, they have not been detected in U.S. food animals (Mulvey et al., 2003; Frye and Fedorka-Cray, 2007; Bush, 2008; Frye et al., 2008). In addition to intI1 and IncA/C plasmids, other vehicles for resistance in the U.S. include many other plasmids such as IncF, IncH, IncI, and IncP which have also been associated with AR (Lindsey et al., 2009; Folster et al., 2010, 2011; Glenn et al., 2011). For example, the IncI1 plasmids are also known to carry the *bla*_CMY−2_ gene; however, they do not usually carry MDR, are found mostly in *Salmonella* serovar Heidelberg, and are associated with chickens and turkeys rather than cattle (Folster et al., 2010, 2012).

### Sources of human outbreaks of *Salmonella*

Outbreaks of *Salmonella* in the U.S. have been associated with food originating from animal sources such as chicken, eggs, beef, and ground turkey (Scallan et al., 2011). However, in the past few decades there has been an increasing number of *Salmonella* outbreaks that have been associated with unusual sources, such as tomatoes, peanut butter, black and red pepper, and spinach (Boxrud et al., 2010). These outbreaks indicate that *Salmonella* infection is not always associated with food animal products such as meat. It is unclear if non-meat sources of *Salmonella* infections have increased or if public health agency programs such as Food Net, Pulse Net, and NARMS have simply enabled detection and tracing of these outbreaks to their sources (Boxrud, 2010; Boxrud et al., 2010). Few outbreaks have been associated with antimicrobial resistant *Salmonella*; however, an exception to this was a 2011 outbreak connected with ground turkey (Folster et al., 2012). A strain of *Salmonella* Heidelberg resistant to ampicillin, gentamicin, streptomycin, and tetracycline, encoded by *bla*_TEM−1_, *aac(3)-IIa, aadA1, ant(3″)-Ia, and tet*(A) on a IncI1 plasmid was detected (Folster et al., 2012).

The wide variety of outbreak sources and variability in genetics causing AR indicates resistance in *Salmonella* is multifaceted, and the trend of unusual food sources and antimicrobial resistant outbreaks may become more prevalent in the future. Additionally, it is also likely that AR elements found mostly outside the U.S. will eventually be found in U.S. food animals. For example, ESBLs have already been found in U.S. human infections and it can be expected to eventually find them in the animal population (Bush, 2008). Surveillance and research of AR in *Salmonella* is a global priority and an ongoing effort will need to continue to follow these trends and identify the mechanisms leading to resistance. Data collected by these studies will improve our understanding of resistance, how it develops, how it spreads, and what can be done to prevent infections in humans and animals.

## Escherichia coli

### Overview of commensal bacteria

Much less studied and understood is the influence exerted by non-pathogens on the acquisition, transfer, and persistence of AR in pathogens sharing the same environment. The intestinal tract of humans and animals are inhabited predominately by Gram-negative and Gram-positive anaerobes such as *Bacteroides* and *Bifidobacterium*. However, many other genera of bacteria including enterococci and *Escherichia coli* are also present. Transient pathogenic colonizers such as *Streptococcus* and *Neisseria* are also present (Salyers et al., 1995). Both indirect and direct evidence exists that AR can be transferred between different genera in the intestinal tract (McConnell et al., 1991; Salyers et al., 1995; Netherwood et al., 1999; Avrain et al., 2004). Furthermore, total numbers and types of bacteria in the intestinal tract can be altered by antimicrobial treatment which may influence the transfer of resistance (McConnell et al., 1991). *E. coli* and enterococci are two commensals of particular interest because they are ubiquitous in animals and the environment and are used most often in bacterial source tracking (Scott et al., 2002; Hassan et al., 2007). They are also known to harbor resistance genes on plasmids, transposons, and integrons (Johnson and Nolan, 2009; Hegstad et al., 2010).

### Commensal and pathogenic *E. coli*

The recognition of *E. coli* as a principal commensal intestinal bacteria and the relative ease of isolating *E. coli* has led to its adoption as a sentinel organism for fecal contamination, particularly as applied to water safety. Those strains which ferment lactose were included in a “coliform bacteria” group of enterobacteria, and coliform enumeration is commonly used to indicate the microbiological safety of food products and water (Blood and Curtis, 1995). However, this simple method cannot be used to identify enteric pathogens nor provide the population data necessary for identifying sources of contamination (Leclerc et al., 2001).

Research has shown that the enteropathogenic *E. coli* (EPEC) are a virulent subgroup possessing a complex array of disease-producing genetic factors absent from commensal strains (Spears et al., 2006). In the 1980's, a pathogenic variant of *E. coli* was recognized as the etiologic agent of potentially fatal hemorrhagic intestinal and hemolytic uremic illnesses (Paton and Paton, 1998; Karch et al., 1999). These toxigenic *E. coli* have acquired Shiga-like toxin genes (Sandvig, 2001; Schmidt, 2001) as a key component of their virulence profile and are termed Shiga toxin-producing *E. coli* (STEC). The STEC were found to kill Vero cells in culture which led to the name verocytotoxigenic *E. coli* (VTEC); they are also categorized as enterohemorrhagic *E. coli* (EHEC). While EPEC and STEC strains share a number of characteristics, the STEC are not simply EPEC that generate a cytolytic toxin (Kaper, 2005). The EPEC appear to be exclusively human pathogens and remain a significant cause of infant diarrheal disease, especially in developing countries. In contrast, the STEC are niche-specific gut commensals in certain animals, particularly ruminants.

The STEC serotype O157:H7 from cattle has become the paradigm for this pathotype (Rasmussen and Casey, 2001; Renter and Sargeant, 2002), but other significant serogroups are emerging (Bettelheim, 2007). Controlling outbreaks of human disease due to contamination of food products by STEC sources is an important food safety issue (Olsvik et al., 1991; Mainil and Daube, 2005; Erickson and Doyle, 2007).

In food animals, *E. coli* has been documented as the etiological agent of many different diseases (Prescott, 2000b). In cattle, *E. coli* infections of the gastrointestinal tract, skin, the genital and urinary tract, the musculoskeletal system, the central nervous system, and the cardiovascular system have been reported (Prescott, 2000b). Some infections such as septicemia and meningitis are often fatal. Colibacillosis in both swine and poultry is also a major cause of mortality in these two groups of food animals (Prescott, 2000b).

### Overview of antimicrobial resistance in *E. coli* isolated from animals

Since all types of *E. coli* acquire AR and are commonly associated with many different animal and environmental sources, they are typically included in AR surveillance studies (Erb et al., 2007). Resistance to aminoglycosides, cephalosporins, fluoroquinolones, and sulfonamide antimicrobials is widespread in *E. coli* isolated from animals and humans and may compromise treatment efficacy since drugs of these classes are frequently used to treat Gram-negative infections (Hammerum and Heuer, 2009). In particular, the prevalence of resistance to clinically important 3rd and 4th generation cephalosporins mediated by extended-spectrum β-lactamases (ESBLs) has serious implications for human and veterinary medicine (Shah et al., 2004a,b; Paterson and Bonomo, 2005; Li et al., 2007).

Multi-drug resistance in *E. coli* is also a concern (Hawkey and Jones, 2009). Resistance in *E. coli* is often mediated by plasmids that encode AR genes (Carattoli, 2009; Johnson et al., 2011a). These extra-chromosomal MGEs are readily acquired by *E. coli* thereby conferring resistance to one or more antimicrobials. The potential threat to human health from multiple drug resistant pathogenic *E. coli* such as STEC acquired from food products may pose a serious yet underestimated food safety risk (Rasko et al., 2011). It is therefore essential to improve our limited knowledge of the basis for the increasing drug resistance of commensal and pathogenic *E. coli*. Since resistance plasmids can be transferred between strains of the same species or different genera (such as *Salmonella* or *Klebsiella*), such exchanges may serve to establish reservoirs of resistance genes in animals and the environment (Su et al., 2008). Furthermore, it should also be noted that there are differences in resistance levels of *E. coli* (including MDR strains) depending on the age of the host. Past studies have shown that younger animals such as cows and swine harbor more resistant *E. coli* than older animals (Langlois et al., 1988; Khachatryan et al., 2004; Berge et al., 2005). It has been suggested that this observation could be due to a higher fitness of the resistant isolates in the intestine of the animals or that the intestine of the younger animals may be more readily colonized by the resistant isolates as compared to the older animals (Langlois et al., 1988; Khachatryan et al., 2004; Berge et al., 2005). Many of the same resistance phenotypes and genotypes presented for *Salmonella* in the previous section are also present in *E. coli*; therefore, AR in *E. coli* will focus on resistance and resistance genes of specific interest in *E. coli* (Table 3).

### Antimicrobial resistance mechanisms in *E. coli* isolated from food animals in the U.S.

#### Aminoglycosides

Since *E. coli* induced diarrhea often occurs in food animals, several aminoglycosides are used in treatment including neomycin for calves, gentamicin in swine, and apramycin in both calves and swine (Prescott, 2000a). Gentamicin is also used for treating septicemia in cattle. Resistance to aminoglycosides is mediated in *E. coli* by genes from all three classes of aminoglycoside modifying enzymes [AAC, ANT, and APH; (Ramirez and Tolmasky, 2010)]. Several acetyltransferases including type II 3-*N-aceyltransferase*, type IV 3-*N*-aceyltransferase, and a type VI 3-*N*-aceyltransferase have all been described in *E. coli* isolated from animals and are located on plasmids (Hedges and Shannon, 1984; Chaslus-Dancla et al., 1987; Johnson et al., 2006a,b, 2011a). Type IV 3-*N*-aceyltransferase was first detected in bovine *E. coli* in France in 1984 and conferred resistance to gentamicin and apramycin. Type II 3-*N*-aceyltransferase was identified a short time later also from bovine isolates. This enzyme was different from the previously described type IV 3-*N*-aceyltransferase which only mediated resistance to gentamicin. A 241-kb plasmid was detected in avian pathogenic *E. coli* isolates (APEC) in 2006 which encoded resistance to gentamicin via *aac(3)-VI* and streptomycin via *aadA* (an adenyltransferase), and additionally conferred resistance to potassium tellurite, silver nitrate, copper sulfate, tetracycline, benzylkonium chloride, and sulfisoxazole (Johnson et al., 2006b). The dissemination of these genes is most likely due to their location on plasmids most of which are capable of mobilization within different *E. coli* strains and to other bacteria as well (Johnson et al., 2006b).

#### β-lactams and cephalosporins

β-lactam and cephem antimicrobials are well-tolerated and present few side-effects in food animals, which make them useful for treatment of disease (Prescott, 2000c,d,g). They have been used to treat *E. coli* infections in poults as well as diarrhea caused by *E. coli* in both neonatal calves and swine. Use of these antimicrobials in food animals has been controversial because they are also very useful in human medicine and represent some of the most critically important antimicrobials used in humans. Resistance to β-lactams and cephems in *E. coli* isolated from U.S. animals has been reported (Li et al., 2007). As reported by NARMS, resistance to β-lactams in *E. coli* isolated from chickens has remained below 26% between the years 2000–2009 (http://www.ars.usda.gov/SP2UserFiles/Place/66120508/NARMS/NARMS2009/Table3C.pdf). Resistance to amoxicillin-clavulanic acid, cefoxitin, ceftriaxone, and ceftiofur for 2009 was 12.4, 11.4, 11.5, and 9.5%, respectively. Resistance to ampicillin ranged from 20% in year 2000 to 19.8% in year 2009 with the highest resistance reported in year 2006 at 25.6%.

Resistance genes detected in *E. coli* isolated from animals are not markedly different from those reported in *Salmonella* from animals (see previous section, Tables 2, 3) with one exception. *E. coli* harbors an endogenous *ampC* gene located on the chromosome, whereas this gene is absent in *Salmonella* (Li et al., 2007). Expression of the “classic” AmpC β-lactamase is not inducible due to the absence of a regulatory gene and is produced at low-levels in the wild-type strains. In contrast, hyper-producers of AmpC β-lactamase have been detected that confer resistance to ampicillin and some cephalosporins (Li et al., 2007). Several factors that influence hyper-production of β-lactamase have been described and many of the hyper-producers have mutations in the promoter region of *ampC* (Siu et al., 2003). A possible homolog to the “classic” *ampC* in *E. coli* has recently been identified in *S.* Typhimurium, but only shares 11% identity with the “classic” *ampC* β-lactamase (McClelland et al., 2001). The ability of this gene to confer functional β-lactam resistance in *S.* Typhimurium has not been demonstrated.

#### Folic acid pathway

Because they have been in use for a long time, resistance to sulfonamides is widespread. Over 50% of *E. coli* isolated from poultry samples by the NARMS program was resistant to sulfisoxazole in year 2009. Conversely, resistance to the combination drug trimethoprim-sulfamethoxazole in poultry *E. coli* was only 7% in year 2009 (http://www.ars.usda.gov/SP2UserFiles/Place/66120508/NARMS/NARMS2009/Table3C.pdf). Resistance to the sulfonamides can be conferred by chromosomal mutations or by resistance genes, *sul1*, *sul2*, and *sul3*, which results in antimicrobial-resistant variants of the target enzymes as described in the *Salmonella* section and Tables 2, 3 (Skold, 2001). Widespread dissemination of the resistance genes by plasmids, integrons, or insertion elements is evidenced by detection of sulfonamide resistance in *E. coli* in humans and food animals in both the U.S. and Europe (Hammerum and Heuer, 2009; Frye et al., 2011; Johnson et al., 2011a; Lindsey et al., 2011b; Glenn et al., 2012). Several mechanisms of resistance to trimethoprim have been identified, including efflux pumps, impaired drug penetration, and mutation in the target enzymes (Skold, 2001). But, as seen for sulfonamide resistance, trimethoprim resistance genes (*dfr*) located on plasmids or transposons-encoding variants to dihydrofolate reductase are predominant.

#### Phenicols

Chloramphenicol is banned for use in U.S. food animals, and resistance is typically low (less than 5% for years 2000–2009 in *E. coli* from poultry from the NARMS program). Several resistance genes have been described that confer resistance to chloramphenicol (White et al., 2000). The plasmid-mediated chloramphenicol acetyltransferase (*cat*) gene is the most common mechanism (Table 3) (Glenn et al., 2012). A non-enzymatic resistance gene encoding an efflux system, *cmlA*, has also been identified in *E. coli* plasmids. A third chloramphenicol resistance gene, *flo*, is also plasmid-encoded and confers resistance to chloramphenicol and florfenicol, a fluorinated structural analog of thiamphenicol and chloramphenicol. In the U.S., florfenicol was approved for use in cattle in 1996 for treatment of respiratory disease (Keyes et al., 2000). Florfenicol resistance has since been detected in clinically ill cattle and chickens in the U.S. (Keyes et al., 2000; White et al., 2000).

#### Quinolones

Mechanisms of fluoroquinolone resistance in *E. coli* and *Salmonella* have been reviewed (Hopkins et al., 2005). Resistance to fluoroquinolones can be due to decreased permeability of the antimicrobial to the cell, efflux pumps, or mutations in DNA gyrase or topoisomerase genes' QRDR motifs (Hopkins et al., 2005). *E. coli* differs from *Salmonella* in that it readily mutates to become resistant under selective pressure, and will maintain the mutations and resistance without selective pressure as there is no apparent fitness cost associated with maintaining resistance in *E. coli* (Bagel et al., 1999; Giraud et al., 1999, 2003; Hopkins et al., 2005). Plasmids harboring quinolone resistance genes (*qnrA*, *qnrB*, and *qnrS*) have also been described, but have not been detected in isolates from U.S. animals. Enrofloxacin and sarafloxacin were approved for use in poultry production in 1995 and 1996, respectively, but this approval was withdrawn in 2005 (Iovine and Blaser, 2004a,b). However, monitoring showed that only 26 of 14,398 (0.18%) *E. coli* isolated from chickens by NARMS from 2000 to 2009 were ciprofloxacin resistant and genetic screening did not detect any known horizontally exchanged quinolone resistance genes (http://www.ars.usda.gov/SP2UserFiles/Place/66120508/NARMS/NARMS2009/Table3C.pdf).

#### Tetracyclines

Tetracyclines have been used for decades in both human and animal medicine and resistance genes are easily acquired. During years 2000–2009, the lowest percentage of resistance among *E. coli* from poultry was 40.2% in 2007 with the highest level of resistance recorded at 68.4% in year 2000 (http://www.ars.usda.gov/SP2UserFiles/Place/66120508/NARMS/NARMS2009/Table3C.pdf). As described for *Salmonella*, tetracycline acts by inhibiting protein synthesis in the bacterial cell. Although the most common acquired mechanisms of tetracycline resistance are efflux pumps and ribosomal protection, the efflux pumps are more prevalent in *E. coli* (Roberts, 2002). To date, nine genes have been identified in *E. coli* isolates that encode proteins for active efflux of tetracycline and/or its derivatives. These include *tet*(A), *tet*(B), *tet*(C), *tet*(D), *tet*(E), *tet*(G), *tet*(J), *tet*(L), and *tet*(Y) (Roberts, 2002, 2005). Resistance genes identified in *E. coli* isolated from chickens in the U.S. are shown in Table 3 (Frye et al., 2011; Glenn et al., 2012). Two genes which encode proteins for ribosomal protection, *tet*(M) and *tet*(W), have also been detected in *E. coli*. However, the *tet*(M) gene may not have a major role in conferring tetracycline resistance in *E. coli* as it has been reported to only confer low-levels of resistance to tetracycline (Roberts, 2002).

## *Enterococcus* spp.

### Definitions and diseases

Although primarily defined as a commensal organism, *Enterococcus* is a Gram-positive bacterium that has an additional role as an opportunistic pathogen causing infections in both humans and animals (Martone, 1998; Cetinkaya et al., 2000; Kuhn et al., 2000). Enterococci are a common inhabitant of the intestinal tract of humans and animals, but have also been isolated from vegetation, soil, water, and food (Niemi et al., 1993; Svec and Sedlacek, 1999; Muller et al., 2001; Giraffa, 2002). Their presence in the digestive tracts of humans and animals coupled with the available methods that exist for molecular typing of the group of bacteria make them useful as an indicator of fecal contamination (Svec and Sedlacek, 1999; Scott et al., 2005; Layton et al., 2010).

An opportunistic pathogen, enterococci are only second to staphylococci as a leading cause of nosocomial infections, accounting for ~12% of hospital-associated infections yearly in the U.S. (Hidron et al., 2008). The majority of infections are caused by two enterococcal species, *Enterococcus faecium* and *E. faecalis* (Huycke et al., 1998). The enterococci have been implicated in a number of human clinical diseases including endocarditis, bacteremia, and urinary tract infections (Jett et al., 1994; Huycke et al., 1998). The majority of enterococcal infections are associated with devices used in hospital settings such as central-lines and catheters, but they are also a common cause of surgical site infections (Hidron et al., 2008). Complicating treatment of enterococcal nosocomial infections is the tendency of the bacterium to harbor AR genes conferring resistance to antimicrobials, such as vancomycin, used to treat enterococcal infections. Furthermore, enterococci are also able to transfer AR genes and some virulence factors to other members of the intestinal microflora, including pathogenic bacteria which increase the risk of resistant nosocomial pathogens (Murray, 1990; Chow et al., 1993; Wirth, 1994; Hancock and Gilmore, 2000).

In addition to causing infections in humans, enterococci have been indicated in infections in animals including food animals such as poultry and cattle (Martone, 1998; Cetinkaya et al., 2000; Kuhn et al., 2000). In poultry, enterococcal species may change over time in the chicken gut and enterococcal infections in poultry can be caused by any of the species that are commonly found in the intestines of the birds; although infections in poultry are sporadic, they can be lethal. Pulmonary hypertension syndrome, amyloid athropathy, bacteremia, encephalomalacia, neurological disorders, and endocarditis have all been described in poultry associated with infection by *E. faecalis*, *E. durans*, and *E. hirae* (Randall et al., 1993; McNamee and King, 1996; Tankson et al., 2001; Steentjes et al., 2002). In dairy cattle, enterococci are primarily associated with bovine mastitis although enterococcal-induced diarrhea in calves has also been reported (Rogers et al., 1992; Madsen et al., 2000). Staphylococci are a major cause of bovine mastitis, but enterococci were implicated in 2–20% of cases where an etiological agent has been identified (Poutrel and Ryniewicz, 1984; Aarestrup et al., 1995; Sobiraj et al., 1997). The route of transmission of enterococci in bovine mastitis is most likely from the environment to the animal as animal to animal infections have not been reported (Rossitto et al., 2002).

While their role as an opportunistic nosocomial pathogen has been well-documented, their ability to cause food-borne illnesses remains largely unknown. While enterococci have been reported to cause diarrhea in animals, this has not been proven in humans. In humans, vomiting and headaches indicative of food intoxication are believed to be caused by the ingestion of fermented food containing enterococci which have produced biogenic amines (Tham et al., 1990; Gardin et al., 2001; Giraffa, 2002). The safety of using enterococci in food production has not been determined as they may be both beneficial as well as detrimental in food processing. In fermented foods, enterococci are essential in manufacturing fermented milk products such as cheeses due to the specific biochemical traits that they possess. Alternatively, they may also be indicative of food spoilage for fermented meats or unsanitary conditions in other food industries (Giraffa, 2002; Foulquie Moreno et al., 2006). Determination of innate traits of the enterococci such as AR and virulence need to be addressed before the safety of using enterococci in food production can be determined.

### Antimicrobial resistance overview of enterococci

Intrinsic resistance to antimicrobial agents used in hospital settings is a common characteristic of enterococci compared to other bacteria primarily found there (Facklam et al., 2002; Malani et al., 2002). Enterococcal infections caused by antimicrobial resistant isolates, including MDR isolates, are more serious and difficult to treat than those caused by susceptible isolates. Some enterococcal species, particularly *E. faecium*, are inherently resistant to some penicillins; and in the past few years, they have also shown increased resistance to vancomycin, cephalosporins, and aminoglycosides in nosocomial infections (Arias et al., 2010). Vancomycin is often considered the last treatment available in serious, MDR infections in humans (Wilson et al., 1995; Marshall et al., 1998; Boneca and Chiosis, 2003). Newer drugs including daptomycin, linezolid, Quinupristin/Dalfopristin, and tigecycline have been developed recently to combat infections caused by Gram-positive bacteria and appear to be promising in the treatment of infections caused by enterococci (Swaney et al., 1998; Projan, 2000; Hancock, 2005; Shoemaker et al., 2006).

Resistance of enterococci in food animals in the U.S. is very similar to what has been described of enterococci isolated from nosocomial infections. Resistance to aminoglycosides, lincosamides, macrolides, nitrofurans, penicillins, quinolones, streptogramins, tetracycline, and rarely vancomycin has been described in poultry, swine, and cattle (Table 4) (Jackson, 2004a,b, 2005, 2011; Jackson et al., 2007; Donabedian et al., 2010; Hammerum et al., 2010; Frye et al., 2011; Marshall and Levy, 2011). With the exception of daptomycin, resistance to the newer antimicrobials (linezolid and tigecycline) has not been detected in food animals (Jackson, 2011).

**Table 4:** **Antimicrobial resistance genes found in enterococci isolated from U.S. food animals**.

**Antimicrobial class**	**Genes**	**References**
Aminoglycosides	*aac(6′)-Ie-aph(2″)-Ia, aac(6′)-Ii, ant(4′)-Ia, ant(6′)-Ia, aph(2″)-Ic, aph(2″)-Id, aph(3″)-IIIa*	Chow et al., 1997; Aarestrup, 2000a; Jackson, 2004b, 2005
Macrolides	*erm*(A), *erm*(B), *msrC*	Aarestrup, 2000a; Werner et al., 2001; Jackson et al., 2007; Schwaiger and Bauer, 2008
Streptogramins	*vat*(B), *vat*(D), *vat*(E), *vga*(B)	Hammerum et al., 1998; Jensen et al., 1998; Werner and Witte, 1999; Jackson et al., 2007
Tetracycline	*tet*(K), *tet*(L), *tet*(M), *tet*(O), *tet*(S)	Aarestrup, 2000a; Aarestrup et al., 2002; Fard et al., 2011
Vancomycin	*vanA*	Donabedian et al., 2010

### Prevalence and mechanisms of resistance in *enterococcus* isolated from food animals

#### Aminoglycosides

According to available data from NARMS, resistance in enterococci from chickens to gentamicin, kanamycin, and streptomycin ranged from 18.6 to 22.3, 29.2 to 32.1, and 14.3 to 20.7%, respectively (http://www.ars.usda.gov/SP2UserFiles/Place/66120508/NARMS/percent_resistance/ENT-RSummary.pdf). Acquired resistance to aminoglycosides is mediated by enzymes categorized as acetyltransferases, adenyltransferases, or phosphotransferases which modify the antimicrobial and is described above (Chow, 2000). Seven aminoglycoside resistance genes have been detected in enterococci from food animals in both the U.S. and Europe (Table 4). Of those, the bifunctional gene *aac(6′)-Ie-aph(2″)-Ia*, is considered the most important in enterococci as it confers resistance to all aminoglycosides used to treat infections including gentamicin, kanamycin, and streptomycin (Aarestrup, 2000a; Chow, 2000). Alleles of *aph(2″)* including *aph(2″)-Ib*, *aph(2″)-Ic*, and *aph(2″)-Id* confer high-level resistance to gentamicin (MIC ≥ 500 μg/ml). Another allele, *aph(2″)-Ie*, was only recently detected in 2006 in *E. casseliflavus* isolated from a hospitalized patient in China (Chen et al., 2006; Watanabe et al., 2009). The deduced amino acid sequence of *aph(2″)-Ie* was almost 94% identical to the amino acid of *aph(2″)-Id.* Prevalence of these *aph(2″)* alleles in enterococci from both humans and animals remains largely undetermined as some reports suggest that they are present in large numbers of enterococci from humans while data from other studies indicate that they are not as widespread in animals (Chow, 2000; Jackson, 2004b, 2005). These discrepancies may be explained by the recent discovery of most of the genes conferring high-level resistance to gentamicin (Chow et al., 1997; Chow, 2000; Chen et al., 2006). It is possible that while they may exist, they may not yet be as widely disseminated as *aac(6′)-Ie-aph(2″)-Ia.*

Compared to gentamicin, fewer aminoglycoside resistance genes have been described that confer resistance to kanamycin and streptomycin. Kanamycin-resistance can be mediated by either *aph(3″)-IIIa* or *aac(6′)-Ii* both of which have been detected in humans and animals in the U.S. (Chow, 2000; Jackson, 2004b, 2005). The high prevalence of *aac(6′)-Ii* in *E. faecium* is most likely due to its presence on the chromosome of all *E. faecium* isolates in both humans and animals. Similarly, intrinsic resistance to streptomycin also exists in enterococci, but it is not mediated by a foreign gene; ribosomal mutations can occur rendering streptomycin resistance levels ≤100 μg/ml (Chow, 2000). Acquired resistance to streptomycin is encoded by *ant(3″)-Ia* (*aadA*) or *ant(6′)-Ia* (*aadE*). Presently, studies have been performed that have detected multiple aminoglycoside resistance genes in enterococci from human and animal sources (Chow, 2000; Jackson, 2004b, 2005). The number of aminoglycoside resistance genes detected to date suggests that this trend of multiple aminoglycoside resistance will continue.

#### Macrolides, lincosamides, and streptogramins

Macrolide, lincosomide, and streptogramin antimicrobials act by binding to the ribosome and preventing protein synthesis (Roberts, 2004). Macrolides and lincosamides are not used to treat human enterococcal infections; they are used in treatment of other bacterial infections and may be substituted in place of other antimicrobials due to allergic reactions (Clermont and Horaud, 1990). In food animals, both macrolides and lincosamides are used to treat infections or as growth promoters (Prescott, 2000f). Lincosamides are used in combination with an aminoglycoside to treat mastitis in dairy cattle in some European countries (De Oliveira et al., 2000). Similarly, lincosamides were used to treat mastitis in dairy cattle on farms in the U.S. (Jackson, 2011). In swine, both lincomycin and the macrolide tylosin are used to treat swine arthritis, ileitis, erysipelas, pneumonia, and swine dysentery; lincomycin is also used to prevent swine dysentery by medicating herds via their drinking water (Prescott, 2000b). Tylosin is also used as medication in the feed of swine in the U.S. to improve feed efficiency and increase weight gain; this practice was banned by the European Union in 1999 when antimicrobials were no longer used as growth promoters (Aarestrup et al., 2001). The ban on growth promoters in Europe also ended the use of virginiamycin, a streptogramin antimicrobial. Virginiamycin has been used in animal production as a growth promoter in the U.S. for decades; it is used in both poultry and swine to increase feed efficiency and promote growth (McEwen and Fedorka-Cray, 2002). More recently, a human analogue of virginiamycin, Synercid® (Quinupristin/Dalfopristin-Q/D) a combination of streptogramin A and B antimicrobials, was approved for use in treatment of vancomycin-resistant enterococci (VRE) in humans (Hancock, 2005). Although virginiamycin has only been used in animals and Q/D in humans, cross-resistance is of particular concern as streptogramin resistance genes can confer resistance to both antimicrobials.

Resistance to macrolides in human clinical enterococci and enterococci from animal sources in Europe as well as the U.S. has been reported (Collignon et al., 2009). Acquired resistance to the macrolides can be due to alteration of the antimicrobial, pumping of the antimicrobial from the cell, or modification of the target. The most common mechanism appears to be target modification by erythromycin resistance methylase (*erm*) genes, primarily *erm*(B) (Mlynarczyk et al., 2010). The *erm*(B) gene confers cross resistance to macrolide, lincosamide, and streptogramin type B antimicrobials, which is characteristic of the macrolide-lincosamide-streptogramin type B (MLS_B_) phenotype (Roberts et al., 1999).

Other macrolide resistance genes which have been detected in the enterococci include *erm*(A) and *msrC*, which is described as an ATP-binding transporter belonging to the efflux pump family of genes (Roberts et al., 1999; Werner et al., 2001; Roberts, 2004; Schwaiger and Bauer, 2008). For lincomycin resistance, *lnu*(B) is the only resistance gene described thus far for *Enterococcus*. The first lincomycin resistance gene, *lnu*(A), was originally described in the staphylococci; to our knowledge *lnu*(B) has not been detected in enterococci from food animals, but it has been detected in enterococci isolated from companion animals (Jackson et al., 2009).

For streptogramin resistance, resistance to the A component is the sole requirement for resistance to both components of the streptogramin A and B combination (Rende-Fournier et al., 1993; Soltani et al., 2000). Higher levels of resistance to streptogramins have been reported as a result of the presence of resistance genes to each single component. Resistance to the B component (quinupristin) in enterococci is mediated by *erm* genes and *msrC* described above. Resistance to the A component (dalfopristin) in the enterococci is mediated by streptogramin A acetyltransferases encoded by *vat*(B), *vat*(D), *vat*(E), or *vat*(G), ATP-binding transporters encoded by *vga*(B), or hydrolases encoded by *vgb*(A) (Hammerum et al., 1998; Jensen et al., 1998; Werner and Witte, 1999; Hershberger, 2004; Roberts, 2004; Jung et al., 2010). The presence of resistance genes to streptogramin A and B antimicrobials differs slightly between Europe and the U.S. Previously, *erm*(B), *msr*C, *vat*(D), and *vat*(E) had all been identified in enterococci from humans, animals, and the environment in Europe; three of the four genes [*erm*(B), *msr*C, and *vat*(E)] had also been identified in enterococci in the U.S. (Hershberger, 2004). The fourth gene, *vat*(D), was only recently found in *E. faecium* and *E. hirae* isolated from chicken carcass rinsates in the U.S. (Jackson et al., 2007). Two newly identified streptogramin resistance genes, *vat*(G) and *vga*(D), were also recently characterized in *E. faecium* isolated from the stool of a healthy human from Korea (Jung et al., 2010). Both genes were detected together on the same plasmid, but have yet to be detected in the U.S. Two additional streptogramin A resistance genes, *vat*(B) and *vga*(B), encoding an acetyltransferase and ATP binding transporter, respectively, were identified in *E. gallinarum* from chicken carcass rinsates in 2008 in the U.S. (Jackson et al., 2008). Both *vat*(B) and *vga*(B) were originally described in a clinical isolate of *S. aureus* from France (Allignet and El Solh, 1995, 1997). Although those genes were located on a plasmid in both *S. aureus* and *E. gallinarum*, plasmid pIP1633 from *S. aureus* is conjugal whereas the *E. gallinarum* plasmid containing *vat*(B) and *vga*(B) is not (Jackson et al., 2008). This was the first report of these genes in *Enterococcus* worldwide. Additional resistance mechanisms to the streptogramin antimicrobials may exist although they have yet to be identified and characterized (McDermott et al., 2005). Streptogramin resistance in *E. faecalis* is typically not reported due the presence of an intrinsic *E. faecalis* species-specific gene, *lsa* (lincosamides-streptogramin A; Singh et al., 2002).

#### Tetracyclines

Tetracycline or the semisynthetic derivative, doxycycline, is used in food animals mainly in treatment of respiratory infections (Mathers et al., 2011). Doxycycline has been used rarely to treat VRE infections in humans possibly due to the high numbers of antimicrobial resistant clinical isolates (Landman and Quale, 1997; Matsumura and Simor, 1998). Overall, tetracycline resistance in enterococci from both humans and animals is widespread and has been previously reviewed (Roberts, 2005). Resistance to tetracycline in enterococci is largely due to ribosomal protection or efflux of the antimicrobial. The most common tetracycline resistance gene is *tet*(M) which encodes proteins for ribosomal protection. The location of this gene in enterococci includes the chromosome, conjugal transposons, Tn916, as well as conjugal plasmids all of which may account for its prevalence. Two additional genes, *tet*(O) and *tet*(S) also confer resistance to tetracycline via ribosomal protection and have been detected in enterococci from food animals (Aarestrup et al., 2002).

The *tet*(L) gene is the most frequently detected tetracycline efflux gene in the enterococci (Bentorcha et al., 1991; Platteeuw et al., 1995). Like *tet*(M), it has also been localized on the chromosome and plasmids in enterococci. A second tetracycline efflux gene, *tet*(K), has also been described in *Enterococcus* (Roberts, 2005; Fard et al., 2011). Previously, a sixth tetracycline resistance gene, *tet*(U) was detected in *E. faecium*, but recent reports suggest that *tet*(U) does not confer resistance to tetracycline in enterococci, but may instead be part of a gene encoding a replication initiator protein (Caryl et al., 2012). New tetracycline derivatives, glycylcyclines, have recently been developed; the first of these is tigecycline (Projan, 2000). For enterococcal infections, tigecycline has been approved for treatment of complicated skin and skin structure infections and complicated intra-abdominal infections caused by vancomycin-susceptible *E. faecalis*. The safety and efficacy of this drug is still being evaluated as reports suggest that there may be an increased risk of mortality when using tigecycline (Yahav et al., 2011). Tigecycline is not approved for use in food animals.

#### Vancomycin

Glycopeptides such as vancomycin bind to peptidoglycan cell wall components and inhibit further synthesis of the bacterial cell wall resulting in their antimicrobial effect. In the U.S., neither vancomycin nor the glycopeptide-related compound, avoparcin, has been approved for use in food animals. Until the European ban on use of growth promoters in food animals, avoparcin was used in European countries for growth promotion (Aarestrup and Seyfarth, 2000; Aarestrup et al., 2000, 2001). The differences in the use of glycopeptide antimicrobials in food animal production in the two regions most likely account for the differences in glycopeptide resistance observed in food animals, but not in humans. In Europe, vancomycin resistance has been found in humans, animals, and the environment (Werner et al., 2008). In contrast, vancomycin resistance in the U.S. was confined to humans where VRE was a leading cause of MDR healthcare-associated infections (Hidron et al., 2008). In a study of healthcare-associated infections conducted by the U.S. CDC, the percentage of VRE in the U.S. healthcare system (33%) was higher than that in Europe and the rest of North America (range 13–28%; Hidron et al., 2008). More specific, the percentages of vancomycin-resistant *E. faecium* pathogenic isolates were also higher in the U.S. (80%) compared to France (0.8%) and Italy (24%). *E. faecium* is more likely than other enterococcal species to harbor vancomycin resistance (Donabedian et al., 2010).

The mechanisms conferring resistance to vancomycin have been previously reviewed (Courvalin, 2006). Briefly, the predominant vancomycin-resistance gene, *vanA*, is an acquired resistance gene and confers resistance to vancomycin and teicoplanin. This gene encodes an enzyme that offers an alternative pathway for peptidoglycan cell wall synthesis that circumvents the obstruction created by glycopeptide antimicrobials bound to the cell wall components. The *vanB* gene is also acquired, but confers resistance to vancomycin only. Both genes have an inducible phenotype and can be located on the chromosome or plasmids. Conversely, *vanC* (*vanC1*, *C2*, or *C3*), an intrinsic gene localized to the chromosome in either *E. casseliflavus*, *E. gallinarum*, or *E. flavescens*, mediates lower levels of resistance (MIC 2–32 μg/ml) to vancomycin only. Several new vancomycin-resistance genes have recently been identified. These include *vanD*, *vanE*, *vanG*, *vanL*, *vanM*, and *vanN* (Courvalin, 2006; Boyd et al., 2008; Xu et al., 2010; Lebreton et al., 2011). Both *vanD* and *vanM* encode D-Ala-D-Lac ligase while *vanE*, *vanG*, *vanL*, and *vanN* encode D-Ala-D-Ser ligase. In addition to the modified target, all of the genes identified to date can be distinguished from each other based upon a number of characteristics including whether they are acquired or intrinsic, the level of resistance to vancomycin and/or teicoplanin, the expression of the resistance (constitutive or inducible), the location of the resistance operon, and the ability of the genes to transfer to other enterococci (Courvalin, 2006). Although the *van* gene cluster organization of *vanM* is most similar to that of *vanD*, the two genes are characteristically different. While *vanD* confers intermediate resistance to vancomycin and teicoplanin, is located on the chromosome and is not transferable by conjugation, *vanM* confers high-level resistance to both vancomycin and teicoplanin, is located on a plasmid, and is transferable (Courvalin, 2006; Xu et al., 2010; Nilsson, 2012). Vancomycin genes *vanE*, *vanG*, and *vanL* confer low level resistance to vancomycin and susceptibility to teicoplanin (Courvalin, 2006; Xu et al., 2010; Nilsson, 2012). All three genes have inducible resistance and are located on the chromosome, but are not mobile. The newest vancomycin-resistance gene, *vanN*, confers resistance to vancomycin only and is the only D-Ala-D-Ser ligase gene that is transferable by conjugation (Lebreton et al., 2011). Until recently, vancomycin-resistance in food animals had not been observed in the U.S.; the first report of vancomycin-resistance in food animals in the U.S. was published in 2010 in which vancomycin-resistant *E. faecium* (MIC ≥ 256 μg/ml) were isolated from swine in Michigan (Donabedian et al., 2010). Those isolates contained *vanA* located on the Tn1546 transposon as previously described for enterococci containing *vanA*. The origin of *vanA* in the Michigan swine samples remains undetermined. No other vancomycin-resistance genes have been identified in the U.S.

## Conclusions

Globally, resistance to antimicrobials appears to be increasing in commensal and environmental isolates as well as in pathogens including foodborne pathogens. This may cause difficulty in treating human and animal infections in the future and merits further surveillance and analyses. *Salmonella* isolated from human and animal infections and also from healthy animals in the U.S. have acquired additional resistance to antimicrobials since the 1990's. Resistance to some antimicrobials appears to have moderated in recent years; however, monitoring will need to be maintained to determine if this trend will continue. In addition, resistance to a number of antimicrobials has also been detected in commensal bacteria and opportunistic pathogens, *E. coli* and *Enterococcus*, isolated from food animals in the U.S. There is also evidence that these and other commensal organisms may serve as reservoirs for AR genes and may transfer these to *Salmonella* and other pathogens. Compounding resistance concerns, international travel and trade makes it likely that resistance mechanism found in other areas of the world may eventually be found in the U.S., as seen for the emergence of ESBLs or vancomycin resistance. The recent identification of the *bla*_MDM−1_ gene in *Salmonella* isolated from a human in the U.S. presents the possibility of a virtually untreatable *Salmonella* infection and underscores these concerns (Savard et al., 2011). Research is ongoing to determine the sources of AR in animals and humans, and the results from these studies will enable an understanding of the complex events behind AR. Analysis of these results may aid in the development of practices that will prevent resistance or slow its spread and thus reduce its impact on human and animal health.

### Conflict of interest statement

The authors declare that the research was conducted in the absence of any commercial or financial relationships that could be construed as a potential conflict of interest.
